# Extract from *Cucurbita pepo* improves BPH symptoms without affecting sexual function: a 24-month noninterventional study

**DOI:** 10.1007/s00345-022-04036-w

**Published:** 2022-05-27

**Authors:** Gerit Theil, Michael Richter, Matthias Schulze, Tilo Köttig, Brigitte Patz, Stefan Heim, Yvonne Krauß, Miroslav Markov, Paolo Fornara

**Affiliations:** 1 University Clinic and Outpatient Clinic for Urology, Medical Faculty of Martin Luther University Halle-Wittenberg, Halle (Saale), Germany; 2 Coordination Center for Clinical Studies/Trial, University Medicine Halle (Saale), Halle (Saale), Germany; 3 Markkleeberg, Germany; 4 Hettstedt, Germany; 5 Gaeufelden, Germany; 6 Omega Pharma Deutschland GmbH, Herrenberg, Germany; 7 Halle (Saale), Germany

**Keywords:** Lower urinary tract symptoms, Prostatic hyperplasia, Phytotherapy, IPSS, Quality of life, Sexual health

## Abstract

**Purpose:**

To assess the symptoms, quality of life and sexual well-being in patients with lower urinary tract symptoms due to benign prostatic hyperplasia LUTS/BPH treated with pumpkin seed soft extract (PSE) in routine practice.

**Methods:**

This noninterventional study included 130 men treated for up to 24 months. The International Prostate Symptom Score (IPSS) and related quality of life, Aging Males’ Symptoms Scale (AMS), and International Index of Erectile Function (IIEF-5) were recorded. Descriptive statistical methods were applied. The mean with 95% confidence interval (CI) was calculated for the primary end point (change in IPSS after 12-month treatment).

**Results:**

Analysis at 12 months included 83 patients [mean (SD) age 65.2 (8.7) years and IPSS (15.6 (3.4), IPSS-QoL 3.4 (0.9)]. AMS and IIEF-5 indicated mild or mild to moderate disorder regarding sexual well-being and erectile dysfunction, respectively. After 12 months, the mean IPSS change from baseline was − 4.7 (95% CI − 5.4 to − 3.9), with 83% (95% CI 65.3 to 84.1) and 53% (95% CI 42.3 to 63.7) of the patients achieving reductions by at least 3 and 5 points, respectively. The proportion of patients with IPSS-QoL below 3 points (mostly satisfied) was 11% (9/83) at baseline and rose to 62% (51/83) and 73% (40/55) at 12 and 24 months, respectively. AMS and IIEF-5 scores did not indicate a negative impact on sexual function during treatment.

**Conclusion:**

In men with a moderate LUTS suggestive of BPH, a low progression risk and an active sex life, treatment with pumpkin seed soft extract provided symptomatic relief, improved IPSS-QoL, and maintained sexual well-being.

**Trial registration:**

DRKS00010729, June 22, 2016.

**Supplementary Information:**

The online version contains supplementary material available at 10.1007/s00345-022-04036-w.

## Introduction

Lower urinary tract symptoms due to benign prostatic hyperplasia (LUTS/BPH) are a widespread condition affecting quality of life [1]. Furthermore, epidemiological studies report a correlation between LUTS severity and sexual function problems such as erectile dysfunction and reduced ejaculation, irrespective of age [2, 3]. Therefore, the potential impact of medication on sex life has become a focus for patient-shared decision-making for symptomatic relief in patients with low risk of progression [4].

The most frequently prescribed substances for LUTS/BPH, such as alpha-blockers and 5-alpha-reductase inhibitors (5ARIs), can lead to various sexual function disorders depending on the drug class and individual substances [5, 6]. Adverse effects on sex life may even persist or worsen after treatment has been terminated [7]. Consequently, attending physicians should inform their patients about these risks, because sexually active men might not be willing to accept a possible impact on their sexual health [5, 6]. In fact, adherence to treatment is generally low in men with LUTS/BPH, and (sexual) side effects may be a reason [5].

Herbal extracts are considered to provide modest symptomatic relief and to be well-tolerated medications that preserve sexual well-being; however, due to the heterogeneity of the herbal extracts available, most guidelines do not offer a recommendation [8, 9]. Herbal medicines used to treat LUTS/BPH include extracts from saw palmetto fruit, stinging nettle root, African prune tree bark and pumpkin seed. In fact, pumpkin seed has long been used for the relief of overactive bladder symptoms, as acknowledged by the European herbal monograph. Possible relevant compounds of herbal drugs include phytosterols, fatty acids, and lectins [9].

Interestingly, the typical and putatively active phytosterols found in pumpkin seeds are Δ7-sterols (avenasterol, spinasterol), which exhibit structural differences from ubiquitous Δ5-sterols (ß-sitosterol, stigmasterol). Uniquely high concentrations of Δ7-sterols are present in pumpkin seed soft extract capsules [10]. In animal models, pumpkin seed oil inhibits testosterone-induced prostate growth [11, 12] and has beneficial urodynamic effects [13] and anti-inflammatory activity [14]. Oil, soft extract and Δ7-sterols from Uromedic^®^ pumpkin, a registered cultivar of the medicinal pumpkin, has shown anti-androgenic effects in experimental studies [15]. In addition to specific Δ7-sterols, other components in pumpkin seeds may contribute to the pharmacological effects, such as oleic acid with binding affinity toward muscarinic receptors [16] and y-tocopherol with anti-inflammatory properties [17].

Pumpkin seed and its soft extract provide symptomatic relief in men with moderate LUTS/BPH and have good tolerability [18, 19]. One objective of treating such patients should be to improve or stabilize their quality of life, physically, psychologically, and sexually.

Here, we assess the quality of life, with a special emphasis on sexual well-being, in a real-life practice setting of LUTS/BPH patients under treatment with pumpkin seed soft extract up to 24 months.

## Methods

### Study design

This prospective noninterventional study was planned by the University Clinic for Urology, Medical Faculty of Martin Luther University Halle-Wittenberg, approved by the responsible ethics committee and conducted in six private urologist practices. All patients provided written informed consent before participation.

Men aged between 18 and 80 years with moderate LUTS suggestive of BPH (LUTS/BPH) were included if they had symptoms for at least the previous 3 months, were previously untreated and had an IPSS ≥ 13. Patients were treated twice daily with capsules containing 500 mg pumpkin seed soft extract (PSE).^1^

Exclusion criteria were neurogenic or malignant disease, recurrent urinary retention or infection, urolithiasis, any treatment for LUTS or procedure at the prostate or bladder during the past three months, catheterization, or renal impairment. The maximum follow-up period was 2 years.

At baseline, demographic data, concomitant diseases and concurrent medications were recorded.

The following validated questionnaires were used to measure the outcome. The self-administered International Prostate Symptom Score (IPSS) questionnaire was employed to evaluate symptomatic relief [20]. In addition, the patients completed the IPSS-related quality-of-life (QoL), Aging Males’ Symptoms Scale (AMS) [21] and erectile function (IIEF-5) [22] questionnaires. The AMS measures health-related quality of life and symptoms in aging men and reflects psychological, somatic and sexual domains.

The IIEF-5 was developed to diagnose the presence and severity of erectile dysfunction (ED).

All questionnaires were completed at each visit, i.e., at baseline and at 3, 6, 12, and 24 months after treatment initiation. Safety evaluation included records of adverse events at all follow-up visits.

### Outcomes

The primary criterion for effectiveness was the absolute change in the IPSS after 12 months of treatment compared to baseline.

Secondary outcomes included improvement in the IPSS of at least 3 and 5 score points after 12 months as well as changes in the IPSS, IPSS-related QoL, AMS and its sexual subscore (AMS-SEX), and IIEF-5 at all visits during the 24-month observation.

### Statistical methods

All data were collected exploratively by using descriptive statistical methods. The demographic and baseline characteristics of the study subjects are summarized as the mean with standard deviation (SD) and median with range. For the primary end point (absolute change in IPSS after 12 months of treatment), the mean change with 95% confidence interval (CI) was calculated.

Secondary outcomes were analyzed descriptively. No imputation was used for missing total scores at individual visits. For examination of safety, the evaluation included all patients who had taken at least one capsule. Analyses were carried out using SAS version 9.4, and GraphPad Prism software version 9.0.

## Results

### Recruitment and patient follow-up

Between May 2016 and July 2020, 130 patients were enrolled. The cohort analyzed for the primary criterion consisted of 83 patients, i.e., all patients with IPSS values at baseline and at the 12-month follow-up. Supplementary Fig. 1 displays the number of patients per visit and reasons for drop-out.

### Baseline characteristics

The mean (SD) age of patients in the primary analysis cohort (*n* = 83) was 65.2 years (8.7). Their mean PSA value, as based on measurement in 52 patients, was 1.9 (1.3) ng/ml. The mean IPSS at the start of PSE treatment was 15.6 (3.4) (Table 1), indicating moderate symptoms. Regarding quality of life related to urological symptoms, patients recorded “mostly satisfied/mixed: equally satisfied/dissatisfied”, corresponding to a mean (SD) of 3.4 (0.9) (Table 1). Only 10.8% (9/83) of the patients had an IPSS-QoL index of “mostly satisfied”, “pleased”, or “delighted” (2 points and below) (Supplementary Table 1). For AMS and AMS sexual subscores, the mean (SD) values at baseline were 24.0 (9.7) and 7.7 (3.7), respectively, indicating the presence of mild symptoms (Table 1, Supplementary Tables 2 and 3). The AMS psychological and somatic subscores are provided in Supplementary Tables 4 and 5. The mean (SD) of the IIEF-5 was 16.4 (5.7), which signified a mild to moderate degree of erectile dysfunction (Table 1, Supplementary Table 6).

**Table 1:** IPSS, AMS, AMS-SEX, and IIEF-5 during the course of treatment

	Baseline (*n* = 83)	3 months (*n* = 83)	6 months (*n* = 83)	12 months (*n* = 83)	24 months (*n* = 55)
IPSS total, range 0–35					
Mean (SD)	15.6 (3.4)	12.0 (4.0)	10.7 (3.6)	10.9 (3.7)	10.1 (2.7)
Median (min; max)	15.0 [10; 32]	12.0 [3; 25]	11.0 [2; 21]	11.0 [3; 24]	10.0 [3; 17]
Mean change [95% CI]		− 3.6 [− 4.3 to − 2.9]	− 4.9 [− 5.7 to − 4.1]	− 4.7 [− 5.4 to − 3.9]	− 5.1 [− 5.9 to − 4.3]
IPSS-QoL, range 0–6					
Mean (SD)	3.4 (0.9)	2.7 (1.0)	2.4 (0.8)	2.3 (0.7)	2.1 (0.7)
Median (min; max)	3 [1; 6]	3 [0; 5]	2 [1; 4]	2 [1; 4]	2 [0; 4]
AMS total score, range 17–85					
Mean (SD)	24.0 (9.7)	23.4 (8.9)	23.6 (9.1)	23.6 (9.0)	23.5 (8.8)
Median (min; max)	20.0 [17; 59]	19.0 [17; 59]	18.0 [17; 62]	20.6 [17; 59]	17.0 [17; 54]
Mean change [95% CI]		− 0.2 [− 0.9 to 0.4]	− 0.4 [− 1.2 to 0.4]	− 0.5 [− 1.2 to 0.1]	0.0 [− 1.0 to 1.0]
AMS-SEX subscale, range 5–25					
Mean (SD)	7.7 (3.7)	7.7 (3.4)	7.8 (3.7)	7.9 (3.7)	7.9 (3.8)
Median (min; max)	5.0 [5; 19]	5.0 [5; 17]	5.0 [5; 18]	5.0 [5; 17]	5.0 [5; 19]
Mean change [95% CI]		− 0.0 [− 0.5 to 0.5]	0.1 [− 0.2 to 0.4]	0.1 [− 0.2 to 0.5]	0.3 [− 0.2 to 0.7]
IIEF-5 score, range 1–25					
Mean (SD)	16.4 (5.7)	16.6 (6.0)	16.9 (6.0)	16.7 (6.3)	15.7 (7.0)
Median (min; max)	19.0 [1; 25]	20.0 [1; 25]	20.0 [1; 25]	20.0 [1; 24]	20.0 [1; 24]
Mean change [95% CI]		0.2 [− 0.4 to 0.8]	0.5 [− 0.3 to 1.3]	0.3 [− 0.5 to 1.1]	− 0.7 [− 2.0 to 0.6]

*AMS* Aging Male's Symptoms: 17 questions with a 5-point rating scale and scores range from 17 to 85; higher scores indicate a greater negative impact on quality of life as follows: none/little 17–26, mild 27–36, moderate 37–49, severe 50–85 [21]

*AMS-SEX *AMS sexual subscore, 5-item sexual domain of the *AMS*; scores range from 5 to 25

*IIEF-5* abridged International Index Of Erectile Function (5 questions) with a 5-point scale; scores range from 1 to 25 to specify the degree of erectile dysfunction (ED) as follows: severe 1–7, mild 8–11, mild to moderate: 12–16, mild: 17–21, no ED (> 21) [22]

### Improvement in LUTS

**Fig. 1:**
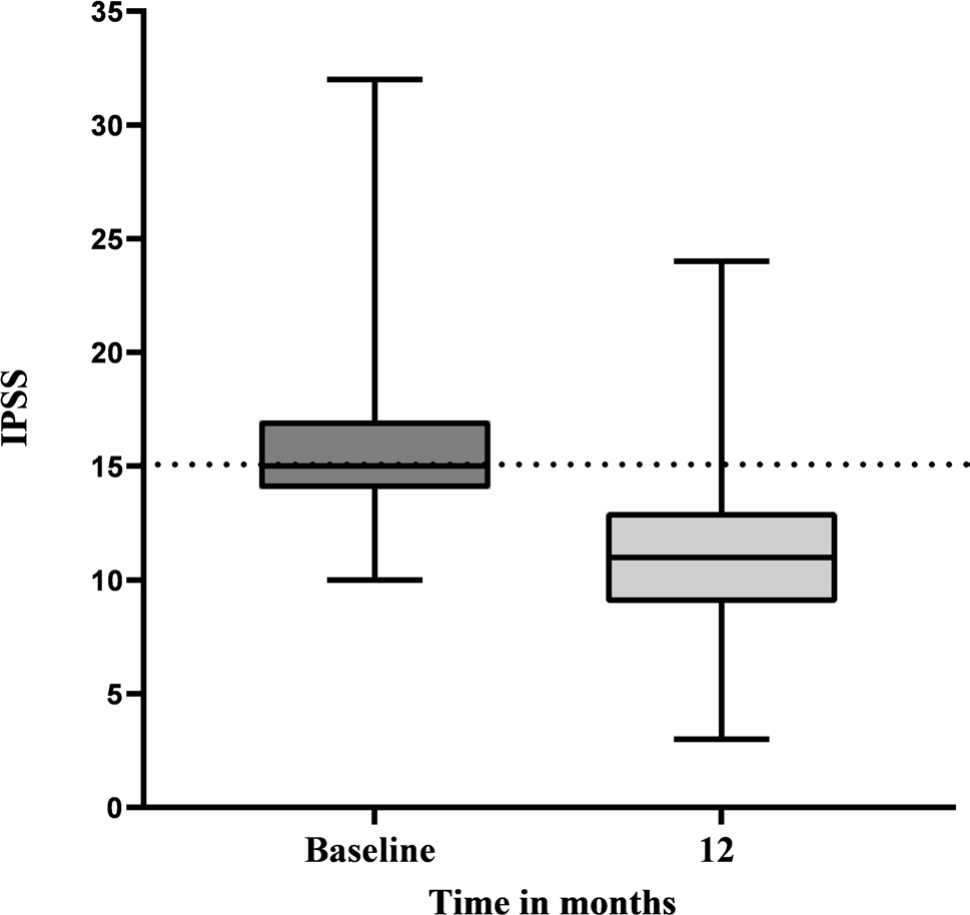
Change in the IPSS over 12 months of treatment, with PSE (*n* = 83) displayed as the mean with minimum and maximum IPSS

The IPSS continuously decreased to a mean (SD) of 10.9 (3.7) at 12 months (Table 1, Fig. 1). The mean change from baseline was − 4.7 (95% CI − 5.4 to − 3.9).

After 12 months, the proportions of patients who had achieved an IPSS reduction by at least 3 and 5 points vs. baseline were 83% (95% CI 65.3 to 84.1) and 53% (95% CI 42.3–63.7), respectively, corresponding to 62 and 44 patients of the 83 analyzed.

### Quality of life and sexual function

IPSS improvement resulted in a relative IPSS-related QoL improvement compared to the baseline of 0.7 (3 months), 1.0 (6 months), 1.1 (12 months), and 1.3 (24 months) (Table 1). At the individual patient level after 3, 6, 12 and 24 months, 43/4 (of 83 patients), 54/0 (of 81), 59/2 (of 83), and 44/1 (of 55), respectively, rated their IPSS-related QoL as being better/worse than that at baseline. For the remaining patients, the score value was unchanged at the respective visit compared to baseline.

**Fig. 2:**
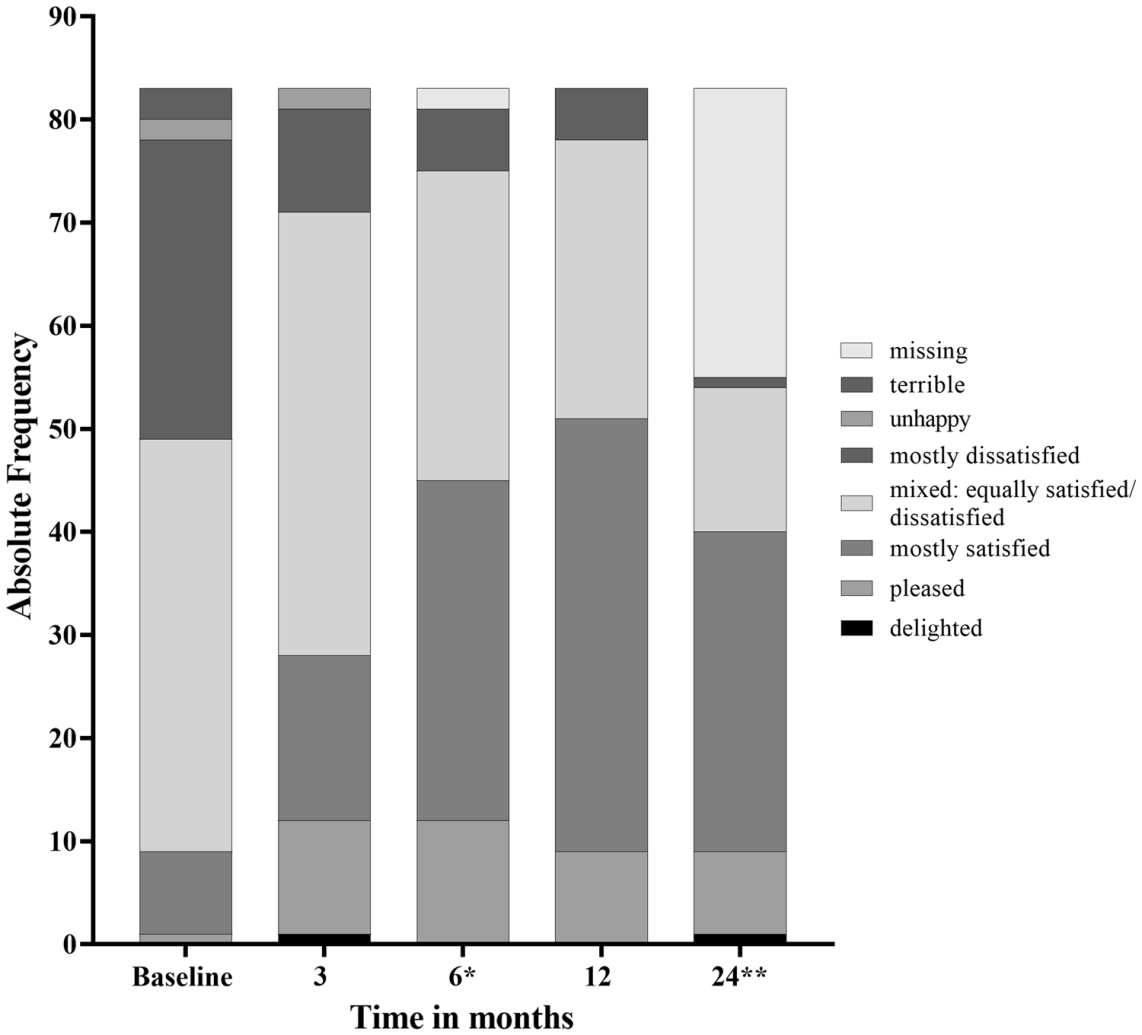
Change in IPSS-related quality of life (QoL) for all study visits (*n* = 83). IPSS-related QoL for all study visits according to the question “If you were to spend the rest of your life with your urinary condition the way it is now, how would you feel about that?” ( primary analysis set, *n* = 83; *missing values for two patients after 6 months. **Treatment was continued for up to 24 months in 55 patients (Fig. 1). Data are presented in Supplementary Table 1

The proportion of patients with an IPSS-QoL index of at least "mostly satisfied “ (2 points and below) increased from 10.8% (9 of 83 patients) at baseline to 61.4% (51 of 83 patients) after 12 months and to 72.7% (40 of 55 patients) after 24 months (Fig. 2, Supplementary Table 1).

Relevant differences from baseline over the course of treatment was not observed for the mean scores for the sexual health questionnaires AMS, AMS-SEX, and IIEF-5 did not show (Table 1, Supplementary Tables 2–6).

### Adverse events

Overall, a total of 16 adverse events were reported in 13 of the 130 patients. However, a causal relationship to the treatment was not found for any of them.

## Discussion

In this study, the symptoms, quality of life, and sexual well-being of 130 patients with LUTS/BPH treated with pumpkin seed soft extract (PSE) were followed up for a maximum period of 2 years in a real-life setting.

The cohort analyzed for the primary criterion consisted of 83 patients treated for at least 12 months. At 12 and 24 months, the IPSS was reduced by 4.7 and 5.1 points compared to baseline in 83 and 55 of the patients available for analysis, respectively. Our results show a stronger effect than that demonstrated by Roehrborn et al. at the 1-year follow-up in their randomized, double-blind study comparing finasteride plus tadalafil with finasteride plus placebo; in that study, three-quarters of the patients achieved an IPSS decrease of at least 3 points and thus perceptible symptom relief [23].

In general, comparisons with previously published studies must be approached with skepticism because IPSS improvement varies greatly between studies; even with placebo, LUTS/BPH patients can achieve a clinically significant IPSS reduction [24]. Another aspect contributing to diverse outcomes is that the magnitude of the IPSS decrease depends on the initial score [25].

Nonetheless, our results suggest a good effectiveness of PSE compared to other phytotherapeutics used to treat LUTS/BPH. The mean reduction in the IPSS of 4.7 points in our investigation is well over the 3.2-point mean reduction found in the real-life practice TRIUMPH study after 12 months of phytotherapy (*Serenoa repens* or *Pygeum africanum*) [26] or the 3.0-point mean reduction in the more recent EVOLUTION European registry study [8].

Moreover, it is noteworthy that the change in the IPSS achieved after 12 months of treatment with PSE was greater than the average effects of placebo in studies investigating plant extracts (− 3.6) and 5ARIs (− 3.4) and, though less pronounced, also greater than the placebo response observed in studies with alpha-blockers and 5ARI/AB combinations (both − 4.3) [27].

Consistent with previous findings [19], relief of LUTS/BPH under treatment with PSE resulted in better IPSS-QoL scores. The proportion of patients who felt at least "mostly satisfied” (IPSS-QoL < 3) increased to 61% and 73% after 12 and 24 months, respectively, from only 11% initially. Furthermore, our results show a mean IPSS-QoL difference of 1.2 (24 months), which is similar to the mean response of 1.1 points observed with tamsulosin or dutasteride [28], and higher than the mean change of 0.9 points observed in men treated with various other phytotherapeutics [8]. This also confirms the benefit of PSE regarding QoL related to LUTS. Moreover, during treatment over two years, the extract was well tolerated, with no impact on the patients' sexual health monitored using the AMS, especially the AMS sexual subscore [21] and IIEF-5 [22]. In contrast, treatment of LUTS/BPH patients with dutasteride 0.5 mg and tamsulosin 0.4 mg compared to placebo resulted in a significant worsening of Male Sexual Health Questionnaire and sexual activity domain scores [29].

A limitation is that the planned sample size was not obtained in this study. After 4 years and inclusion of 130 patients, the study was prematurely terminated due to little prospect of involving additional patients in an acceptable time. One explanation for this is that phytotherapeutics are prescribed less frequently than synthetic agents. Furthermore, herbal medicine is associated with higher costs for most patients, as herbal products are usually not reimbursed in Germany.

Finally, of the original 130 patients, only 83 (64%) and 48 (37%) remained adherent to PSE treatment at 12 and 24 months, suggesting that financial aspects caused withdrawal from PSE treatment. On the other hand, adherence to any medical therapy decreases over time in LUTS/BPH patients. Indeed, recent reported rates for alpha-blockers and combination therapy at 1 year were just 39.2% and 45.6%, respectively [30, 31]. Compared to this, the adherence rate in our study indicates a good acceptance of PSE by patients who had started it.

During the entire study period, 28 patients switched to synthetic medication (mainly tamsulosin). However, in most of them, the IPSS improved compared to baseline, implying that nonmedical factors may have also been responsible, as opposed to symptomatic progression or an unsatisfactory effect.

## Conclusion

Patients with LUTS/BPH, who typically present in daily medical practice, were monitored under routine practice conditions. The patients had moderate LUTS impairing their health-related QoL, but no relevant impairment of sexual health and were treated with pumpkin seed soft extract for up to 24 months. The IPSS was reduced by at least 3 points in three-quarters of the patients and by 5 points or more in half of the patients after 12 months. Using valid questionnaires, this study shows that long-term treatment with pumpkin seed soft extract has no negative impact on a patient's sexual well-being. PSE may be offered as a long-term treatment for those with a low risk of progression and preference for treatment without impacting sexual health. For future studies, it would be interesting to compare the effect of PSE on symptom reduction and sexual health to alpha-blockers in a randomized trial.

## Electronic supplementary material

Below is the link to the electronic supplementary material.

Supplementary material 1 (DOCX 649 kb)

## Footnotes

1 Brands: GRANU FINK Prosta forte 500 mg (DE), GRANU FINK Prosta kemény (HU), GRANUFINK Prosta forte (AT, CH, NL, UA), and Urostemol Prosta (UK).
