# Correction: Extract from *Cucurbita pepo* improves BPH symptoms without affecting sexual function: a 24-month noninterventional study

**DOI:** 10.1007/s00345-022-04132-x

**Published:** 2022-09-03

**Authors:** Gerit Theil, Michael Richter, Matthias Schulze, Tilo Köttig, Brigitte Patz, Stefan Heim, Yvonne Krauß, Miroslav Markov, Paolo Fornara

**Affiliations:** 1 University Clinic and Outpatient Clinic for Urology, Medical Faculty of Martin Luther University Halle-Wittenberg, Halle (Saale), Germany; 2 Coordination Center for Clinical Studies/Trial, University Medicine Halle (Saale), Halle (Saale), Germany; 3 Markkleeberg, Germany; 4 Hettstedt, Germany; 5 Gaeufelden, Germany; 6 Omega Pharma Deutschland GmbH, Herrenberg, Germany; 7 Halle (Saale), Germany

## Correction: World Journal of Urology (2022) 40:1769–1775 10.1007/s00345-022-04036-w

In the original publication of the article, the authors noticed a mistake in Fig. 2. The correct Fig. 2 is given below.

**Fig. 2:**
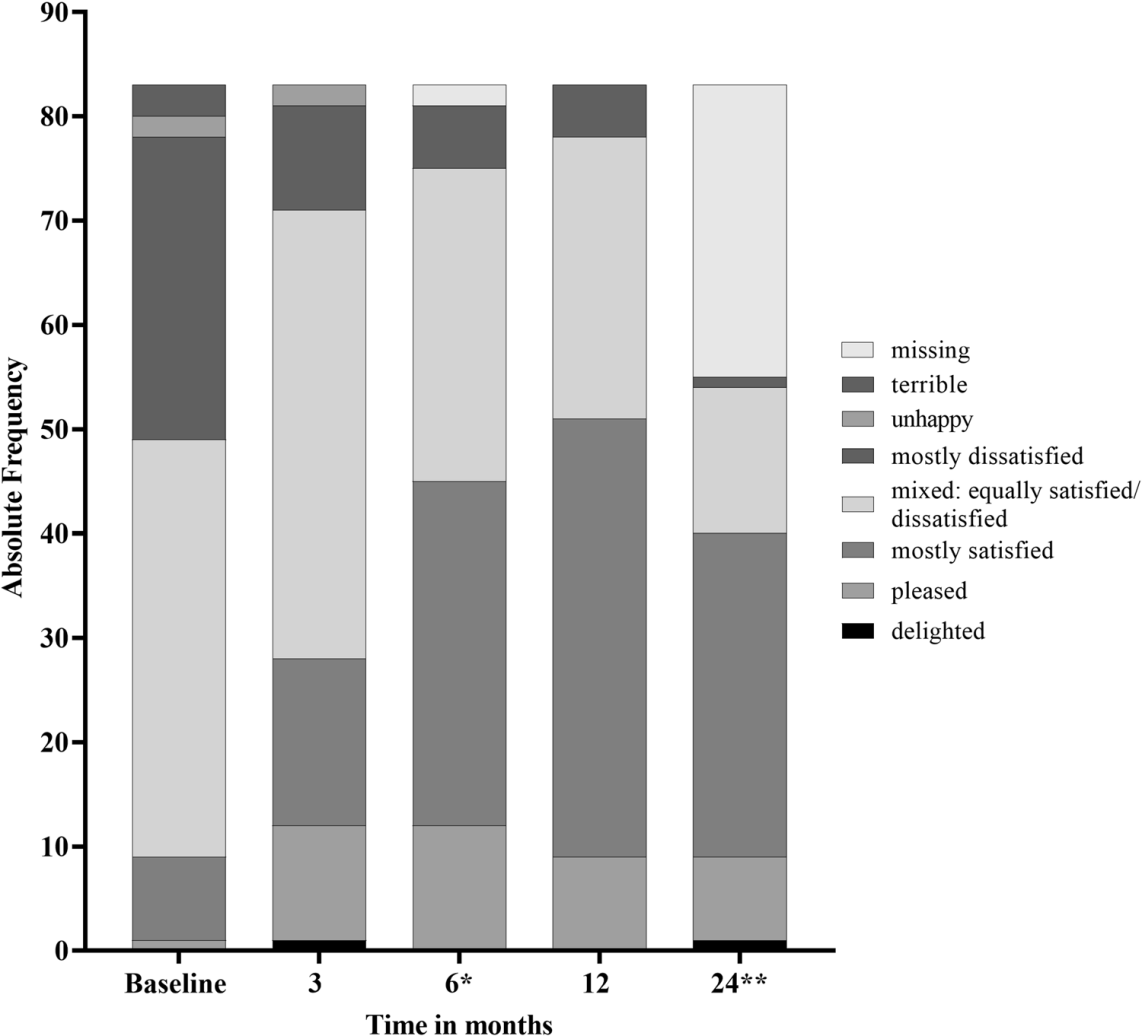
Change in IPSS-related quality of life (QoL) for all study visits (*n* = 83). IPSS-related QoL for all study visits according to the question “If you were to spend the rest of your life with your urinary condition the way it is now, how would you feel about that?” (primary analysis set, *n* = 83; *missing values for two patients after 6 months. **Treatment was continued for up to 24 months in 55 patients (Fig. 1). Data are presented in Supplementary Table 1

The original article has been corrected.

