# Supplementary material for: Extract from Cucurbita pepo improves BPH symptoms without affecting sexual function: a 24-month noninterventional study
Source: World J Urol. 2022 May 27;40(7):1769–75. doi: 10.1007/s00345-022-04036-w (PMC9236993; doi:10.1007/s00345-022-04036-w)
Supplement: Supplementary file 1 — Supplementary material 1 (DOCX 649 kb) [file 345_2022_4036_MOESM1_ESM.pdf]

Supplementary data

Extract from *Cucurbita pepo* improves BPH symptoms without affecting sexual function: a 24-month noninterventional study

Gerit Theil · Michael Richter · Matthias Schulze · Tilo Köttig · Brigitte Patz · Stefan Heim · Yvonne Krauß · Miroslav Markov · Paolo Fornara

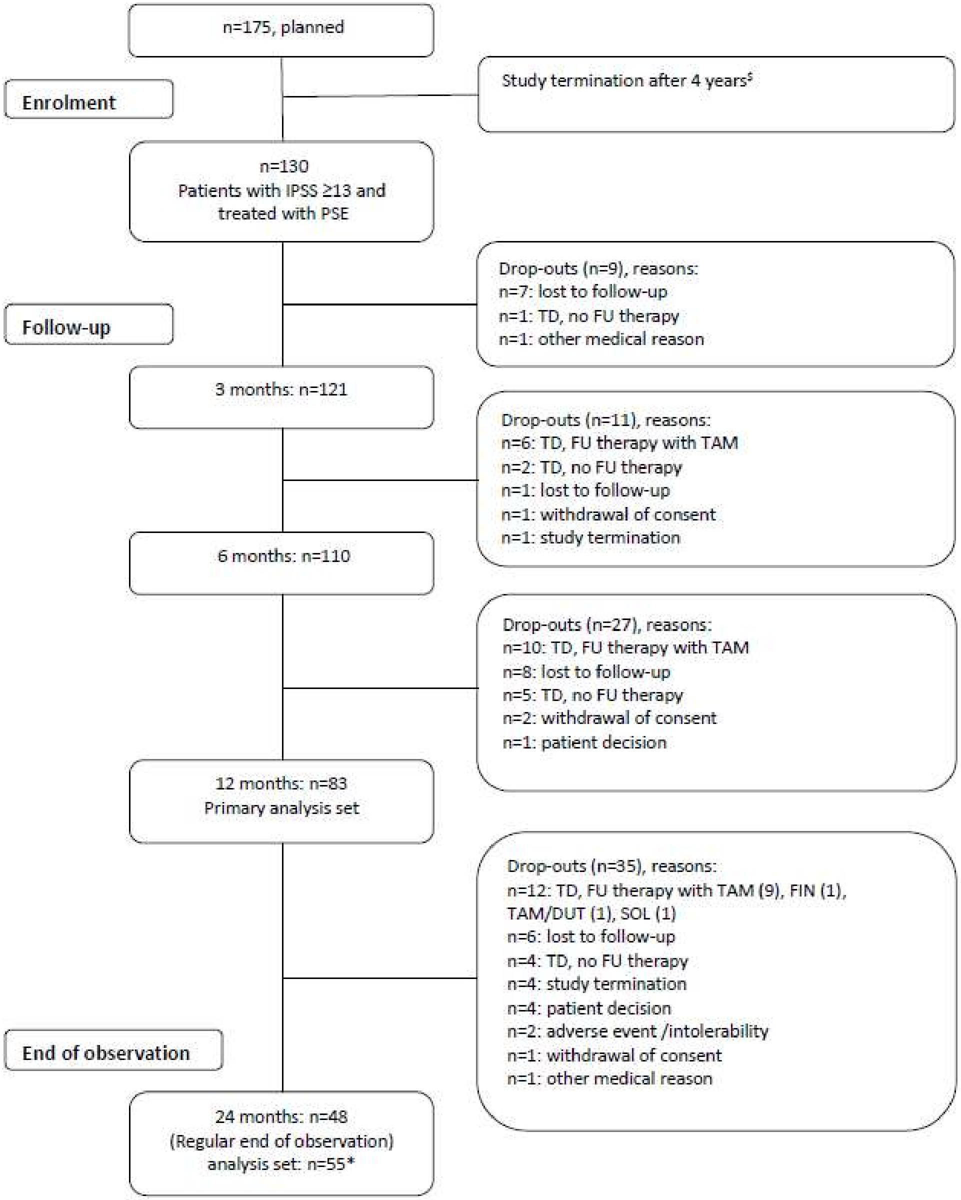

Supplementary Figure 1
Patient flow: The number of patients at baseline, 3, 6, 12 and 24 months and reasons for drop-out.

Footnotes: § Study was terminated due to slow patient recruitment. * The 24-month analysis included the data of 55 patients (48 with regular end at 24 months and 7 with last visits deviating from the 24-month schedule).

Abbreviations: PSE, pumpkin seed soft extract; TD, treatment discontinuation; FU, Follow-up; TAM, Tamsulosin; FIN, finasteride; TAM/DUT, combination tamsulosin/dutasteride; SOL, solifencain. Overall, 28 patients switched to FU with a synthetic drug. At the time of drop-out, the IPSS was lower, unchanged, and maximum 2 points higher than at baseline in 21, 3, and 3 of these patients, respectively. No follow-up IPSS was available for 1 patient.

Supplementary Table 1

IPSS-related Quality of life (QoL) through 24-month treatment with pumpkin seed soft extract

IPSS-QoL, proportion of patients							
**Score**	**0** Delighted	**1** Pleased	**2** Mostly satisfied	**3** Mixed*	**4** Mostly dissatisfied	**5** Unhappy	**6** Terrible
**Baseline (n=83)**							
**Number of patients (%)**	0 (0.0)	1 (1.2)	8 (9.6)	40 (48.2)	29 (35.0)	2 (2.4)	3 (3.6)
**Cumulative: n (%) <3 and ≥ 3 points#**		9 (10.8)			74 (89.2)		
**3 months (n=83)**							
**Number of patients (%)**	1 (1.2)	11 (13.3)	16 (19.3)	43 (51.8)	10 (12.0)	2 (2.4)	0 (0.0)
**Cumulative: n (%) <3 and ≥ 3 points#**		28 (33.8)			45 (66.2)		
**6 months (n=81)**							
**Number of patients (%)**	0 (0.0)	12 (14.8)	33 (40.8)	30 (37.0)	6 (7.4)	0 (0.0)	0 (0.0)
**Cumulative: n (%) <3 and ≥ 3 points#**		45 (55.6)			36 (44.4)		
**12 months (n=83)**							
**Number of patients (%)**	0 (0.0)	9 (10.8)	42 (50.6)	27 (32.5)	5 (6.0)	0 (0.0)	0 (0.0)
**Cumulative: n (%) <3 and ≥ 3 points#**		51 (61.4)			32 (38.5)		
**24 months (n=55)**							
**Number of patients (%)**	1 (1.8)	8 (14.5)	31 (56.4)	14 (25.5)	1 (1.8)	0 (0.0)	0 (0.0)
**Cumulative: n (%) <3 and ≥ 3 points#**		40 (72.7)			15 (27.3)		

IPSS-QoL, IPSS related quality of life question to be rated on a 7-point scale (If you were to spend the rest of your life with your urinary condition the way it is now, how would you feel about that?); * mixed: equally satisfied and dissatisfied.
QoL ≥ 3 points is considered as bothersome

Supplementary Table 2
AMS total score through 24-month treatment with pumpkin seed soft extract

A Absolute scores per visit

Visit	N	mean	SD	min	Q1	median	Q3	max
**Baseline**	83	24.0	9.7	17	17.0	20.0	28.7	59
**3 months**	82§	23.4	8.9	17	17.0	19.0	28.0	59
**6 months**	83	23.6	9.1	17	17.0	18.0	28.0	62
**12 months**	82§	23.6	9.0	17	17.0	20.6	27.0	59
**24 months**	55*	23.5	8.8	17	17.0	17.0	28.0	54

B Changes vs. baseline

Visit	N	mean [95% CI]	SD	min	Q1	median	Q3	max
**3 months**	82§	-0.2 [-0.9 to 0.4]	2.8	-14	0.0	0.0	0.0	11
**6 months**	83	-0.4 [-1.2 to 0.4]	3.7	-16	-1.0	0.0	0.0	12
**12 months**	82§	-0.5 [-1.2 to 0.1]	3.0	-16	-1.0	0.0	0.0	9
**24 months**	55*	0.0 [-1.0 to 1.0]	3.6	-11	-1.0	0.0	0.0	15

AMS, Aging Male's Symptoms: 17 questions with a 5-point rating scale and score range from 17 to 85.
n=83, patients treated for 12 months (primary analysis set); § score missing in 1 patient; *55 patients continued treatment up to 24 months.
Higher scores indicate a greater negative impact on quality-of-life as follows: none/little 17-26, mild 27-36, moderate 37-49, severe 50-85.

Supplementary Table 3
AMS sexual subscore through 24-month treatment with pumpkin seed soft extract

A Absolute scores per visit

Visit	N	mean	SD	min	Q1	median	Q3	max
**Baseline**	83	7.7	3.7	5	5.0	5.0	10.0	19
**3 months**	83	7.7	3.4	5	5.0	5.0	11.0	17
**6 months**	83	7.8	3.7	5	5.0	5.0	10.0	18
**12 months**	82§	7.9	3.7	5	5.0	5.0	11.0	17
**24 months**	55*	7.9	3.8	5	5.0	5.0	10.0	19

B changes vs. baseline

Visit	N	mean [95% CI]	SD	min	Q1	median	Q3	max
**3 months**	83	-0.0 [-0.5 to 0.5]	2.2	-10	0.0	0.0	1.0	5
**6 months**	83	0.1 [-0.2 to 0.4]	1.5	-6	0.0	0.0	1.0	5
**12 months**	82§	0.1 [-0.2 to 0.5]	1.7	-7	0.0	0.0	0.0	7
**24 months**	55*	0.3 [-0.2 to 0.7]	1.7	-4	0.0	0.0	0.0	6

AMS, Aging Male's Symptoms sexual subscore: 5 questions with a 5-point rating scale and score range from 5 to 25.
n=83, patients treated for 12 months (primary analysis set); § score missing in 1 patient; *55 patients continued treatment up to 24 months.
Higher scores indicate a greater negative impact on quality-of-life.

Supplementary Table 4
AMS psychological subscore through 24-month treatment with pumpkin seed soft extract

A Absolute scores per visit

Visit	N	mean	SD	min	Q1	median	Q3	max
**Baseline**	83	6.1	2.5	5	5.0	5.0	6.0	19
**3 months**	82§	6.0	2.3	5	5.0	5.0	7.0	19
**6 months**	83	5.9	2.4	5	5.0	5.0	6.0	22
**12 months**	82§	5.9	2.3	5	5.0	5.0	6.0	19
**24 months**	55*	5.9	2.0	5	5.0	5.0	6.0	14

B Changes vs. baseline

Visit	N	mean [95% CI]	SD	min	Q1	median	Q3	max
**3 months**	82§	-0.0 [-0.2 to 0.2]	1.0	-4	0.0	0.0	0.0	3
**6 months**	83	-0.1 [-0.4 to 0.2]	1.4	-5	0.0	0.0	0.0	5
**12 months**	82§	-0.2 [-0.4 to 0.1]	1.0	-5	0.0	0.0	0.0	3
**24 months**	55*	0.0 [-0.3 to 0.4]	1.3	-5	0.0	0.0	0.0	5

AMS, Aging Male's Symptoms psychological subscore: 5 questions with a 5-point rating scale and score range from 5 to 25. n=83, patients treated for 12 months (primary analysis set); § score missing in 1 patient; *55 patients continued treatment up to 24 months.
Higher scores indicate a greater negative impact on quality-of-life.

Supplementary Table 5
AMS somatic subscore through 24-month treatment with pumpkin seed soft extract

A Absolute scores per visit

Visit	N	mean	SD	min	Q1	median	Q3	max
**Baseline**	83	10.2	4.6	7	7.0	8.0	12.0	26
**3 months**	83	9.8	3.9	7	7.0	7.0	12.0	23
**6 months**	83	9.9	3.9	7	7.0	7.0	12.0	23
**12 months**	82§	9.8	4.0	7	7.0	7.5	11.0	23
**24 months**	55*	9.6	4.0	7	7.0	7.0	11.0	25

B Changes vs. baseline

Visit	N	mean [95% CI]	SD	min	Q1	median	Q3	max
**3 months**	83	-0.5 [-0.9 to -0.1]	2.0	-14	-1.0	0.0	0.0	4
**6 months**	83	-0.4 [-0.9 to 0.1]	2.2	-10	-1.0	0.0	0.0	6
**12 months**	82§	-0.5 [-1.0 to -0.1]	2.0	-9	-1.0	0.0	0.0	6
**24 months**	55*	-0.3 [-0.8 to 0.1]	1.7	-5	-1.0	0.0	0.0	7

AMS, Aging Male's Symptoms somatic subscore: 7 questions with a 5-point rating scale and score range from 5 to 35.
n=83, patients treated for 12 months (primary analysis set); § score missing in 1 patient; *55 patients continued treatment up to 24 months.
Higher scores indicate a greater negative impact on quality-of-life.

Supplementary Table 6
IIEF-5 score through 24-month treatment with pumpkin seed soft extract

A Absolute scores per visit

Visit	N	mean	SD	min	Q1	median	Q3	max
**Baseline**	83	16.4	5.7	1	14.0	19.0	20.0	25
**3 months**	83	16.6	6.0	1	15.0	20.0	20.0	25
**6 months**	83	16.9	6.0	1	15.0	20.0	20.0	25
**12 months**	82§	16.7	6.3	1	16.0	20.0	20.0	24
**24 months**	55*	15.7	7.0	1	13.0	20.0	20.0	24

B Changes vs. baseline

Visit	N	mean [95% CI]	SD	min	Q1	median	Q3	max
**3 months**	83	0.2 [-0.4 to -0.8]	2.7	-11	0.0	0.0	1.0	6
**6 months**	83	0.5 [-0.3 to 1.3]	3.8	-13	0.0	0.0	1.0	17
**12 months**	82§	0.3 [-0.5 to 1.1]	3.7	-13	0.0	0.0	1.0	15
**24 months**	55*	-0.7 [-2.0 to 0.6]	4.8	-15	-1.0	0.0	0.0	15

IIEF-5, abridged International Index of Erectile Function (5 questions) with 5-point scale; score range from 1 to 25 specifying the degree of erectile dysfunction (ED) as follows: severe 1-7, mild 8-11, mild to moderate: 12-16, mild: 17-21, no ED (>21)

n=83, patients treated for 12 months (primary analysis set); § missing value for 1 patients; *55 patients continued treatment up to 24 months.
